# Simultaneous Single‐Position Oblique Lumbar Interbody Fusion and Percutaneous Pedicle Screw Fixation under O‐Arm Navigation for Modified MISDEF Type II Adult Degenerative Scoliosis: Case Series and Surgical Technique

**DOI:** 10.1111/os.14179

**Published:** 2024-08-19

**Authors:** Yan Wang, Shuo Han, Zhu Guo, Chong Sun, Xuexiao Ma

**Affiliations:** ^1^ Department of Spinal Surgery the Affiliated Hospital of Qingdao University Qingdao China

**Keywords:** navigation, oblique lumbar interbody fusion, percutaneous pedicel screw fixation, surgical technique

## Abstract

**Purpose:**

Oblique lumbar interbody fusion (OLIF) has become a popular technique for treating adult degenerative scoliosis (ADS), but traditional OLIF technology often requires repositioning for one‐stage or staged posterior fixation. The objective of this pilot study was to describe the surgical technique of simultaneous single‐position OLIF and percutaneous pedicle screw fixation (OLIF 360) under O‐Arm navigation for modified MISDEF type II ADS.

**Methods:**

Between June 2022 and December 2023, six patients classified as having modified MISDEF type II ADS underwent OLIF 360 assisted by O‐Arm navigation at our institution. Intraoperative blood loss, duration of operation, and complications related to the OLIF 360 procedure were recorded. The preoperative and postoperative spinal pelvic parameters were measured using X‐rays. The accuracy of pedicel screws was recorded in accordance with the modified Gertzbein–Robbins classification on CT. Postoperative MRI was performed to evaluate the indirect decompressive effect. The Japanese Orthopedic Association score for low back pain was used to evaluate surgical outcomes.

**Results:**

Navigated OLIF 360 were performed in six ADS patients with 44 percutaneous pedicel screws and 16 cages placement, including four women and two men. The mean operation time was 160.83 ± 33.23 min, and the mean blood loss was 111.67 ± 39.71 mL. Postoperative spinal pelvic parameters and spinal stenosis degree improved significantly on X‐ray and MRI. All screws were clinically acceptable according to the Gertzbein–Robbins classification, with 92.7% grade A and 7.3% grade B. No serious intraoperative and postoperative adverse events were recorded in all patients. The JOA scores for low back pain of all patients were significantly improved at postoperative 1 month and the final follow‐up.

**Conclusion:**

We report on a case series and describe navigated OLIF 360 in treating modified MISDEF type II ADS patients. Navigation‐assisted OLIF 360 has shown encouraging surgical outcomes with good spinal imbalance correction and indirect decompression.

## Graphical abstract

OLIF 360 technology is used to perform the OLIF procedure and percutaneous pedicle screw fixation in a single position simultaneously. We report on a case series and describe navigated OLIF 360 in treating ADS, which has shown encouraging surgical outcomes with good spinal imbalance correction and indirect decompression.

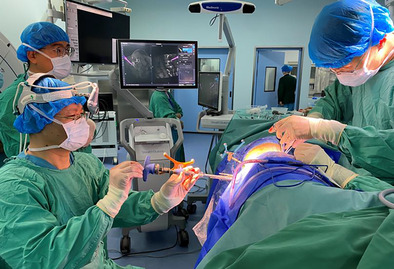

## Introduction

The pathological basis of adult degenerative scoliosis (ADS) is the degeneration of facet joints with asymmetric disc degeneration, resulting in coronal imbalance, which is associated with the reduction of lumbar lordosis or sagittal imbalance, spinal canal stenosis, and lateral vertebral slip.^1^ Oblique lumbar interbody fusion (OLIF) plays an important role in the minimally invasive treatment of ADS, which has the advantages of less trauma and faster recovery than traditional posterior surgery.^2^, ^3^, ^4^ OLIF increases the tension of the ligamentum flavum, annulus fibrosus, and posterior longitudinal ligament to achieve the goal of indirect decompression of the spinal canal and intervertebral foramen.^5^ The lumbar disc is reshaped by OLIF through the expansion of the interbody space, while the Cobb angle or lateral slip is corrected in ADS.^2^ In addition, most of the apical vertebras are in L_2/3_ and L_3/4_ segments in ADS, and the optimal surgical levels of OLIF technology are OLIF 25, which also provides a natural anatomical advantage for OLIF technology in treating ADS. In patients with rigid ADS, the coronal and sagittal balance can be further restored using the OLIF procedure with intervertebral space release. This procedure can improve the lordosis and scoliosis of the lumbar spine, downgrade the curve's apex, and reduce the grade of the posterior osteotomy or fixation segment.^2^, ^6^

OLIF 360 technology is used to perform the OLIF procedure and percutaneous pedicle screw fixation (PPSF) in a single lateral decubitus position.^7^, ^8^, ^9^, ^10^ The navigation‐assisted OLIF 360 guides the procedure from the initial exposure of intervertebral space to discectomy to final cage placement, and the whole procedure is visual and controllable. At the same time, PPSF under navigation provides a three‐dimensional (3D) and accurate trajectory in the decubitus position without the need for repositioning.^8^, ^9^, ^10^ Several studies have demonstrated the effectiveness of traditional OLIF and pedicle screw fixation in the prone position for treating patients with symptomatic ADS.^2^, ^3^, ^4^ However, published literature is lacking about simultaneous single‐position OLIF with PPSF for ADS. In this study, we describe a series of cases to evaluate and analyze the clinical, surgical, and radiographic outcomes of OLIF 360 in treating ADS. This study aimed to (i) describe the indication for OLIF 360 in ADS; (ii) describe the surgical procedures and technical principles of OLIF 360 under navigation in ADS; and (iii) investigate the surgical and radiographic outcome of navigation‐assisted OLIF 360 for patients with ADS.

## Method

Six patients diagnosed with ADS who underwent OLIF 360 assisted by the Stealth Station S8 surgical navigation system (MEDTRONIC, USA) in our institution from June 2022 to December 2023 were included in our study. All six patients were classified as type II deformities according to the modified minimally invasive spinal deformity surgery (MISDEF) algorithm.^11^ Inclusion criteria were: (i) pain or paralysis relieved by rest; and (ii) the absence of facet fusion, extruded disc, bony central canal, or lateral recess stenosis on CT and MRI. Exclusion criteria included prior history of retroperitoneal surgery, high‐grade spondylolisthesis (>grade 2), and severe osteoporosis (T‐score <−3.5). The diagnosis was confirmed by clinical symptoms and imaging findings. All procedures were performed by the same team of experienced surgeons. The details of the six patients are provided in Table 1. The procedures were approved by the ethics committee of the Affiliated Hospital of Qingdao University (approval number: QYFYWZLL 28624). Written informed consent was obtained from all participants.

**TABLE 1:** Patients’ basic information and clinical parameters.

Case No	Sex/Age	OLIF and posterior fixation level	Duration (month)	Op time (min)	Blood loss (mL)	Adverse events	Follow‐up (month)	JOA score for low back pain (max: 29)		
								Preoperative/postoperative (recovery rate)		
									Post 1 month	Final
1	M/75	L_2/3_, L_3/4_, L_4/5_	24	200	150	None	14	8	21 (61.9%)	23 (71.4%)
2	M/65	L_2/3_, L_3/4_	12	120	50	None	12	10	22 (63.2%)	25 (78.9%)
3	F/64	L_2/3_, L_3/4_	6	135	80	None	12	13	25 (75%)	26 (81.3%)
4	F/68	L_2/3_, L_3/4_, L_4/5_	30	200	150	Abdominal bloating and distension.	18	8	23 (71.4%)	25 (81%)
5	F/78	L_2/3_, L_3/4_, L_4/5_	6	160	120	None	22	8	23 (71.4%)	26 (85.7%)
6	F/59	L_2/3_, L_3/4_, L_4/5_	12	150	120	None	20	9	20 (55%)	21 (60%)

OLIF, oblique lumbar interbody fusion.

### Data Collection

Intraoperative observations were recorded for blood loss and duration of operation. Adverse events or surgical complications related to this technique, such as nerve or dural sac injury, endplate fracture, hematoma, vascular injury, incisional infection, transient hip flexion weakness and transient thigh pain or numbness, ureteral injury, symptomatic adjacent segment degeneration, and instrument failure or pseudoarthrosis, were also recorded during the operation and at final follow‐up.

Immediate postoperative X‐ray and CT scans were performed to observe the accuracy of percutaneous screws, which was recorded in accordance with the modified Gertzbein–Robbins classification^12^ (grade A, no breach or deviation; B, breach <2 mm; C, 2 mm < breach ≤ 4 mm; D, breach >4 mm). Screw grade A and B were considered clinically acceptable, and screw grade C and D were defined as in malposition. Immediate postoperative MRI was performed and Schizas classification^13^ was used to evaluate the indirect decompressive effect. CT was performed at the final follow‐up to assess the fusion status. The Japanese Orthopedic Association (JOA) score for low back pain^14^ was used to evaluate surgical outcomes preoperatively and postoperatively at 1 month and at the final follow‐up (average at postoperative 17.4 months and range 12–24 months) to evaluate the clinical outcome.

### Surgical Technique of Navigated OLIF 360 in adult degenerative scoliosis

#### Positioning of the Patient

**FIGURE 1:**
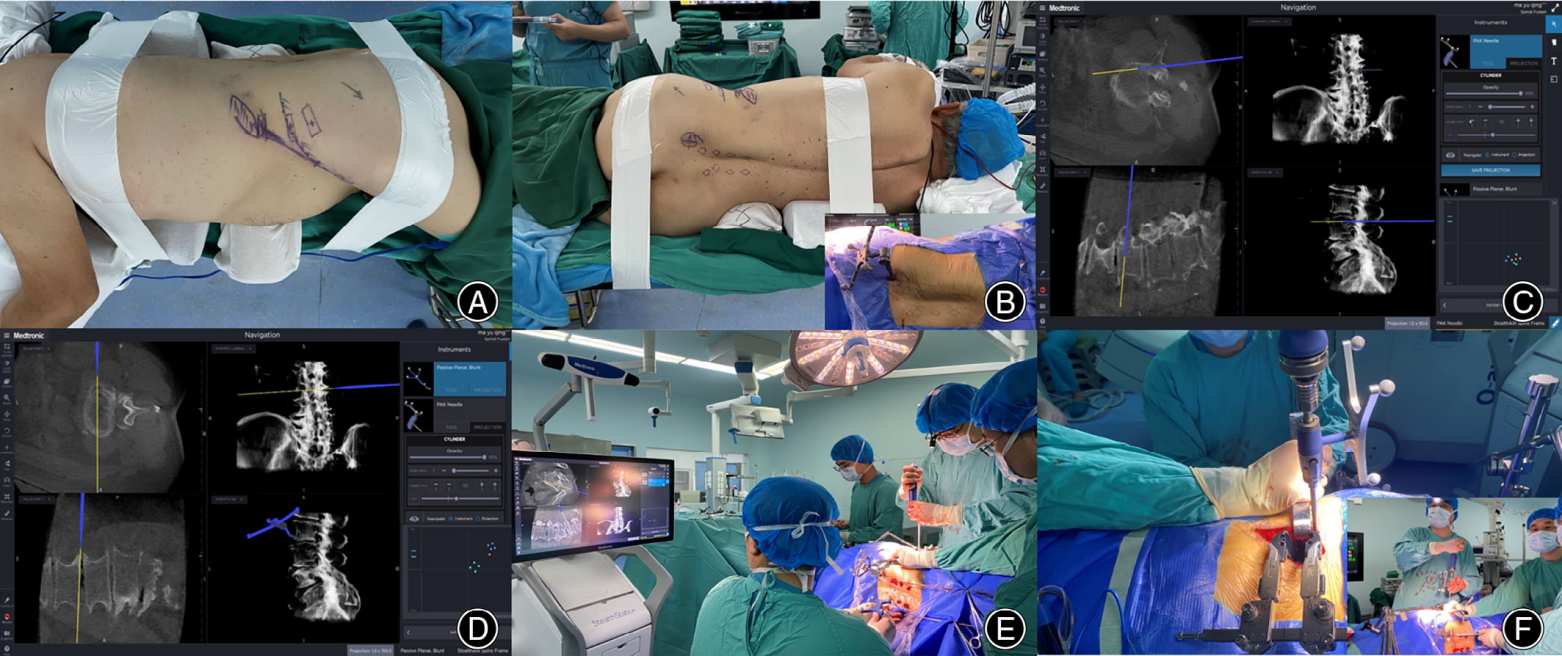
The patient was positioned in right lateral decubitus position (A, B). A reference frame was placed at the posterior superior iliac spine and an O‐arm scan was performed (B). Percutaneous pedicle screw fixation (PPSF) under navigation was performed simultaneously (C). A navigation probe was used to located the disc surface before oblique lumbar interbody fusion (OLIF) retractor placement (D). Navigation‐assisted disc preparation and PPSF were performed simultaneously (E,F).

After endotracheal intubation for general anesthesia, the patient was positioned in a right lateral decubitus position in the carbon fiber bed. The dorsal side of the patient was close to the edge of the bed, and enough space was reserved for the screw placement on the right side. The right lower limb was bent to increase stability. Non‐elastic tapes were used to fix the upper thoracic vertebra below the shoulder and below the iliac crest (across the level of the greater trochanter of the femur, avoiding the posterior superior iliac crest and the range of the OLIF procedure) and to fix a pillow between the lower legs. The angle of the operating bed was adjusted to ensure a true lateral position (Figure 1A, B).

#### Navigation Registration

The navigation camera and screen were placed on the causal side of the patient. The navigated instruments were verified by placing the tip of the navigation tool in the center concavity of the navigation reference frame and ensuring the spheres on the tools and reference frame faced the navigation camera. After disinfection and laying towels, a sterilized bag was attached at the back to collect the possible blood from the screw placement. The posterior superior iliac spine was marked, and the reference frame was placed. The percutaneous nail of the reference frame was positioned at 60° toward the midline and 30° toward the caudal side to avoid blocking the tracker during the operation (Figure 1B). An O‐arm scan was performed for image data acquisition and 3D data were transferred to the stealth station. Intraoperatively, the OLIF procedure and PPSF were performed simultaneously from the segment furthest away from the reference frame. The completion of the preparation of cephalic pedicel screw trajectories was confirmed before disc preparation to avoid the impact of caudal manipulation on the accuracy of proximal screw placement.

#### Navigation‐Assisted Oblique Lumbar Interbody Fusion and Percutaneous Pedicle Screw Fixation

**FIGURE 2:**
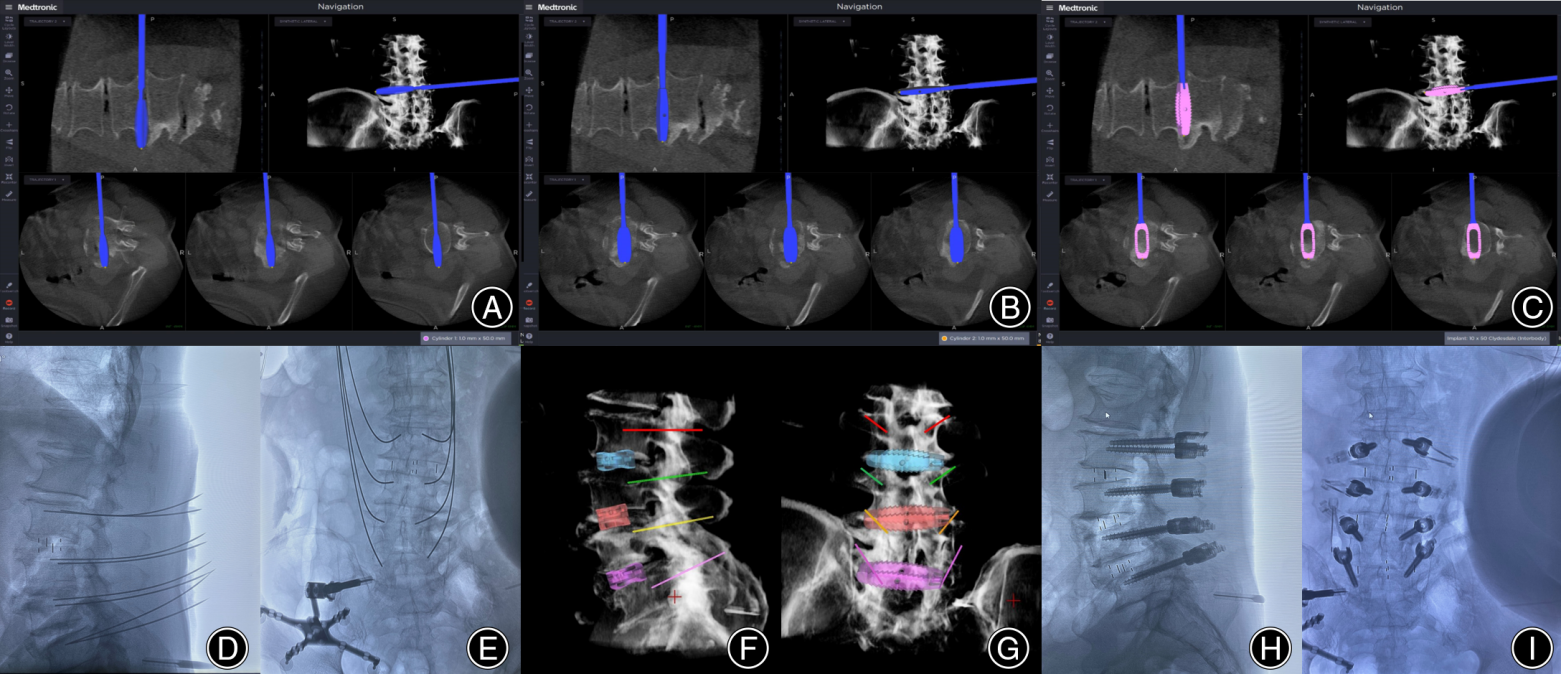
Release the contralateral annulus or osteophyte with a reamer (A). Trialing and oblique lumbar interbody fusion (OLIF) implant placement under navigation were performed (B, C). Guide wires of percutaneous pedicle screw fixation (PPSF) and the final position of OLIF cages and pedicle screws were evaluated on intraoperative X‐ray and navigated three‐dimensional (3D) image (D–I).

First, a navigation probe was used to locate the disc space on the body surface. The OLIF procedure started from the cephalic disc furthest away from the reference frame, and PPSF under navigation was performed simultaneously (Figure 1C). After the blunt separation of abdominal muscles and entering the retroperitoneal space, the retroperitoneal fat tissue was swiped away from the posterior abdominal wall and psoas by a blunt finger. The navigated dilator was docked to the disc surface between the psoas and abdominal major blood vessels (Figure 1D). Sequentially dilatation was performed, and the retractor was placed. The lateral discectomy was performed, and the cartilage endplate was scraped (Figure 1E). With the help of navigation, the reamer was used to penetrate and release the contralateral annulus fibrosus or osteophyte bridge with an orthogonal maneuver (Figures 1F and 2A). Following disc preparation, the navigated templates were used sequentially to distract disc space until adequate disc height and foraminal size were achieved (Figure 2B). Allogeneic bone was placed in the central cavity of cage and the implant was attached to the navigated inserter. The cage was inserted using an orthogonal maneuver under navigational assistance (Figure 2C). The important point was that the OLIF and PPSF procedures started from the cephalic segment, and the OLIF cage was inserted after completing the trajectory of the cephalic pedicle screws because navigational accuracy will decrease if the caudal cage is placed first.

At the same time as the front OLIF operation, the PPSF under navigation was also performed at the cephalic vertebra furthest away from the reference frame. The navigated pedicle access kit (PAK) needle was used to identify the entry point and mark the skin where the screws would be placed. A virtual guide wire was created for the tip of the PAK needle to help with accurate screw placement. After the PAK needle was placed, the guide wire placement and taps were performed in turn. Before the screws are placed, it is necessary to determine the accuracy of the guide wire position by fluoroscopy (Figure 2D,E) or use the non‐guide wire screw placement technology to directly place the screw after self‐drilling tap placement. After the placement of the OLIF interbody cages and screws, a 3D image was generated in the software of navigation system and fluoroscopy was performed to evaluate the final position of internal fixation (Figure 2F–I). Anterior closure and the posterior final rod locking were performed simultaneously.

### Illustrative Cases

#### Case 1

**FIGURE 3:**
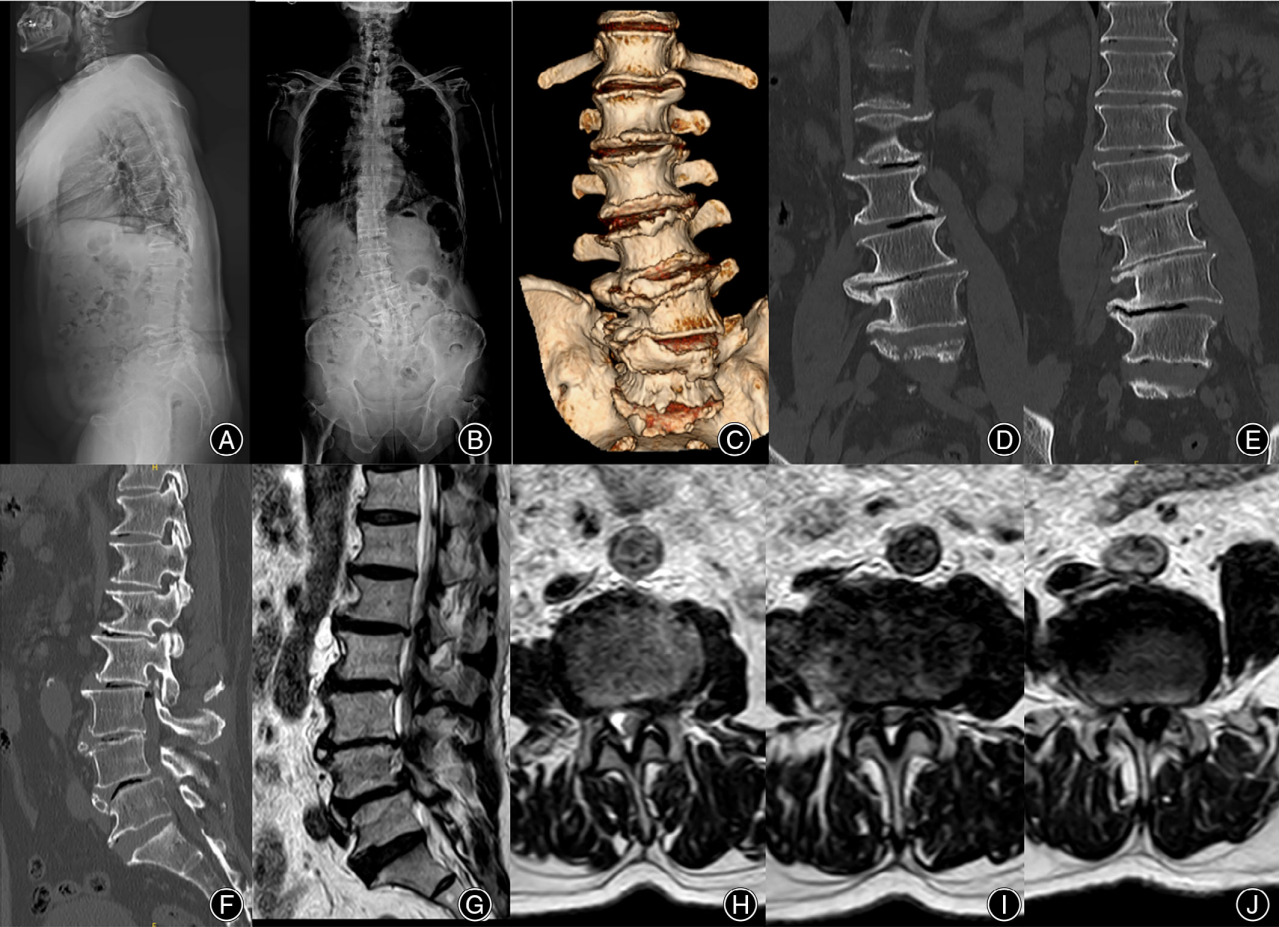
Whole spine X‐rays showed lumbar scoliosis with coronal and sagittal imbalance (A, B). CT showed lumbar degenerative spondylolisthesis (L4), with an osteophyte bridge at the right lateral intervertebral space of L_3/4_ and L_4/5_ (C–F). MRI showed severe spinal stenosis at L_3/4_ and L_4/5_ segments, and Schizas grade was C and D, respectively (G–J).

A 75‐year‐old man presented with low back pain and pain in both hips and lateral thighs for 2 years. He had intermittent claudication for 100 m, with his body leaned forward and normal posture difficult to maintain, which was completely relieved after bed rest. Whole spine X‐ray showed lumbar scoliosis with coronal and sagittal imbalance. CT showed lumbar degenerative spondylolisthesis (L4), with an osteophyte bridge at the right lateral intervertebral space of L_3/4_ and L_4/5_. MRI showed severe spinal stenosis at L_3/4_ and L_4/5_ with Schizas grade C and D, respectively (Figure 3).

**FIGURE 4:**
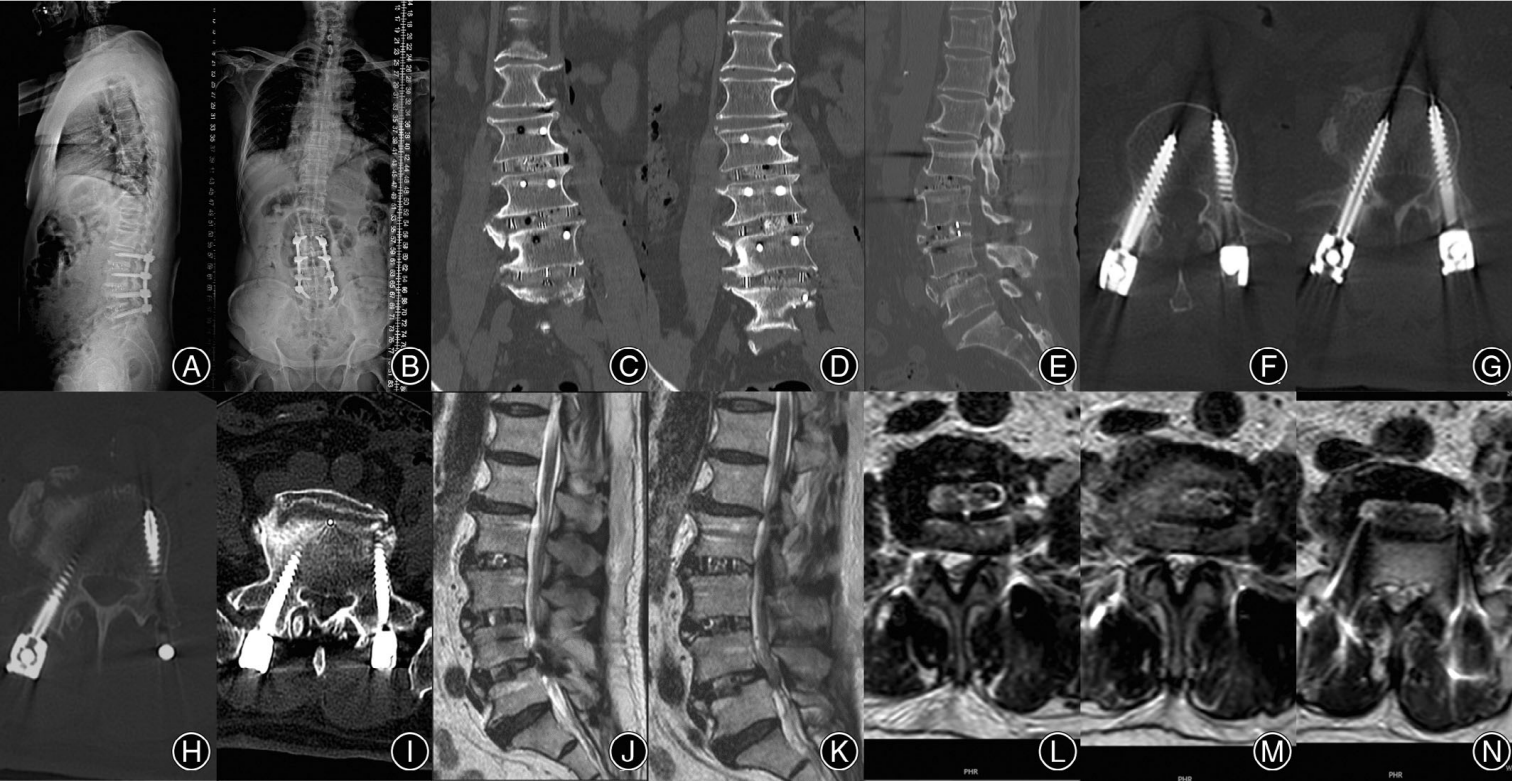
Coronal and sagittal imbalance of the lumbar spine were significantly improved on postoperative X‐ray (A, B). The osteophyte bridge at the contralateral disc space of L_3/4_ and L_4/5_ was released sufficiency (C–E). Postoperative CT showed that pedicles were all grade‐A according to the Gertzbein–Robbins classification and the degree of lumbar spondylolisthesis was significantly improved (F–I). Postoperative MRI showed that the degree of lumbar spinal stenosis was significantly improved, and the Schizas grades of L_3/4_ and L_4/5_ segments were A3 and A4, respectively (J–N).

The patient underwent an OLIF 360 operation with the operative level at L_2/3_, L_3/4_, and L_4/5_. During the operation, 8 mm, 10 mm, and 12 mm reamers were used to fully release the contralateral bone bridge of the L_3/4_ and L_4/5_ before trailing and implant placement. Posterior L_2_ to L_5_ PPSF was performed simultaneously. The coronal and sagittal imbalance of the lumbar spine were significantly improved on the postoperative X‐ray. Postoperative CT showed that pedicle screws were all grade A according to the Gertzbein–Robbins classification, and the degree of lumbar spondylolisthesis was improved significantly. The osteophyte bridge at the contralateral disc space of L_3/4_ and L_4/5_ was also released sufficiency. Postoperative MRI showed that the degree of lumbar spinal stenosis was significantly improved, and the Schizas grades of L_3/4_ and L_4/5_ segments were A3 and A4, respectively (Figure 4).

#### Case 2

**FIGURE 5:**
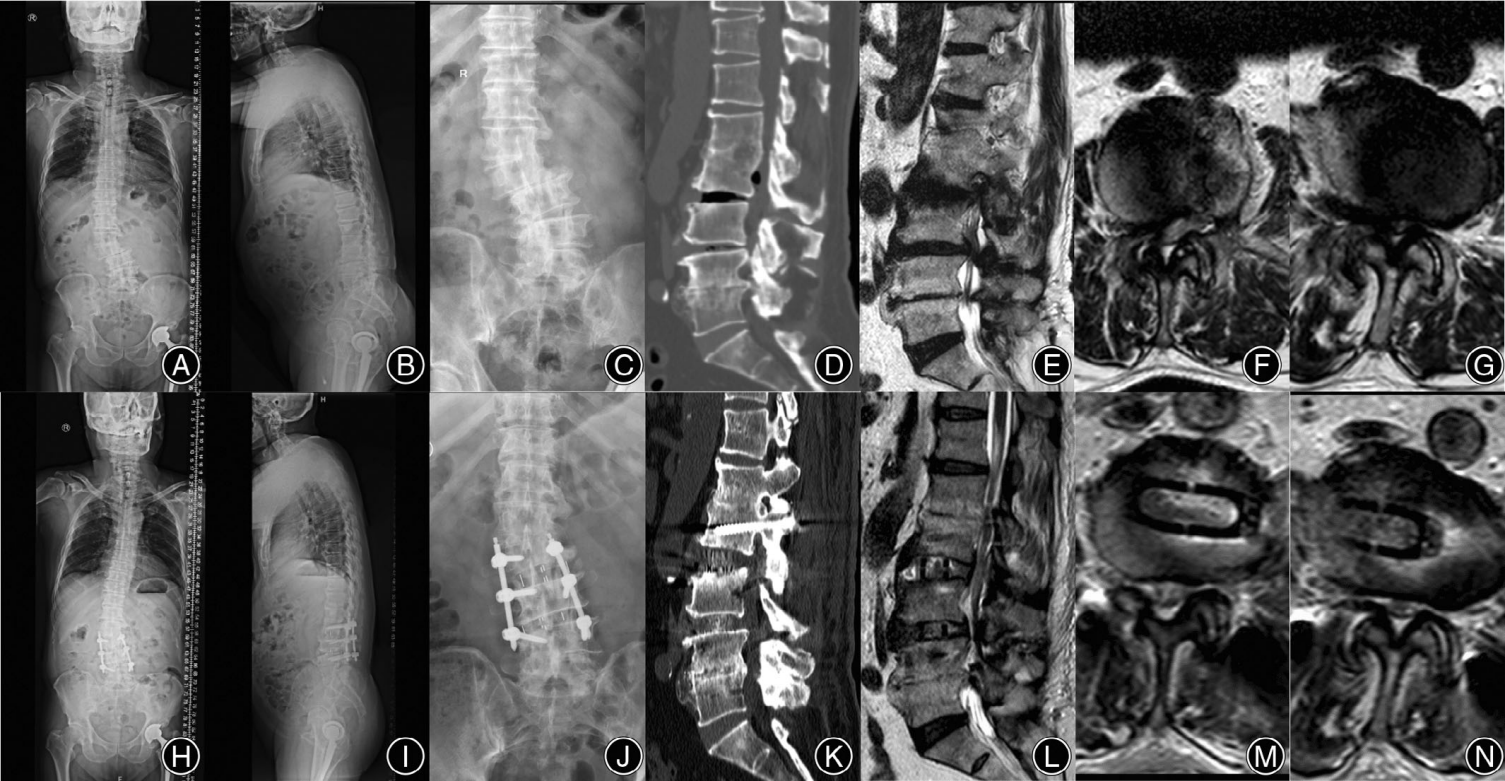
Whole spine X‐ray showed significant L2 lateral slip, reduced lumbar lordosis, and sagittal imbalance (A–C). CT showed spontaneous fusion of L_1/2_ and L_4/5_ interbody spaces and an intradiscal vacuum phenomenon of L_2/3_ and L_3/4_ (D). MRI showed severe spinal stenosis at L_2/3_ and L_3/4_ segments, and the Schizas grade was C and D, respectively (E–G). Postoperative X‐rays showed that the lateral slip of L_2_ and L_3_ vertebras was significantly improved, and CT showed that the height of L_2/3_ and L_3/4_ disc space increased. MRI showed that the spinal canal stenosis degree of L_2/3_ and L_3/4_ were both improved to Schizas grade B (L–N).

A 65‐year‐old man with lumbar disc herniation underwent fenestration discectomy at L_1/2_ and L_4/5_. Twenty years after the first surgery, he had low back pain with numbness of both lower limbs, difficulty with ambulation, and an inability to maintain a forward gaze after walking 50 m, but these symptoms were relieved after bed rest. A whole spine X‐ray showed significant lateral slip of the L2 vertebral body, reduced lumbar lordosis, and sagittal imbalance. CT showed spontaneous fusion of L_1/2_ and L_4/5_ interbody spaces, and intradiscal vacuum changes at L_2/3_ and L_3/4_. MRI showed severe spinal stenosis at L_2/3_ and L_3/4_, and Schizas grade was C and D, respectively (Figure 5A–G).

The patient underwent an OLIF 360 operation, with L_2/3_ and L_3/4_ OLIF and PPSF simultaneously. Postoperative X‐rays showed that the lateral slip of L_2_ and L_3_ vertebra was significantly improved. CT showed that the height of disc space had increased without end plate injury. MRI showed that the central spinal canal stenosis was significantly improved, and the Schizas grades of L_2/3_ and L_3/4_ segments were both improved to grade B (Figure 5H–N).

#### Case 3

**FIGURE 6:**
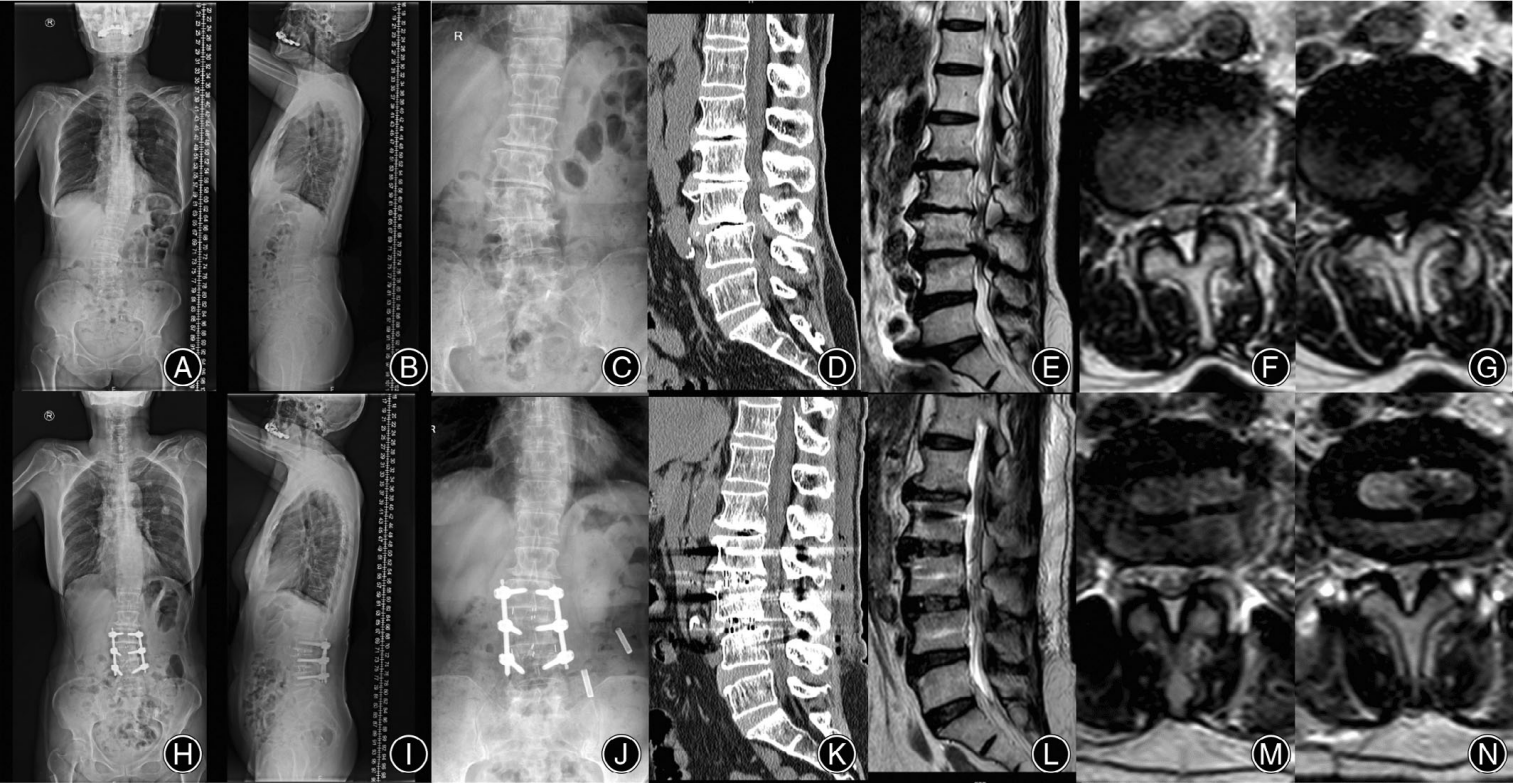
X‐rays showed scoliosis of the lumbar spine, lateral slip of L_3_, and lateral bone bridge at L_2/3_ level (A–C). CT showed L_3_ spondylolisthesis and stenosis at L_3/4_ intervertebral space (D). MRI showed severe stenosis at L_3/4_ with Schizas grade D (E‐G). Postoperative X‐rays showed that lumbar scoliosis was improved (H–J). CT showed that the lumbar spondylolisthesis was improved and the height of the intervertebral space was restored (K). MRI showed that the spinal canal stenosis at L_3/4_ was improved to Schizas grade B (L–N).

An elderly female aged 64, presented with mechanical low back pain and intermittent claudication for 6 months, which was completely relieved after bed rest. X‐rays showed mild scoliosis of the lumbar spine, lateral slip of the L3 vertebral body, and formation of a lateral bone bridge at L_2/3_ level. CT showed L3 spondylolisthesis and significant stenosis at L_3/4_. MRI showed extremely severe stenosis at the L_3/4_ segment with Schizas grade D and obvious cauda equina redundancy at L_2/3_ (Figure 6A–G).

The patient underwent an OLIF 360 operation at L_2/3_ and L_3/4_. The postoperative X‐ray showed that the lumbar scoliosis was improved. CT showed that the lumbar spondylolisthesis was improved, and the height of the intervertebral space was restored satisfactorily. MRI showed that the cauda equina redundancy was significantly improved at L_2/3_, and the spinal canal stenosis at the L_3/4_ segment was improved to Schizas grade B (Figure 6H–N).

## Result

Patients’ basic information and clinical parameters are summarized in Table 1. The mean operation time was 160.83 ± 33.23 min, and the mean blood loss was 111.67 ± 39.71 mL. All six patients’ symptoms were immediately relieved after the surgery. Compared with the preoperative radiological parameters, postoperative values such as sagittal vertical axis, coronal vertical axis, lumbar lordosis, pelvic tilt, sacral slope, and Cobb angle were markedly improved (Table 2). Postoperative MRI showed that the severity of spinal stenosis greatly improved through indirect compression. One patient suffered postoperative abdominal distension, and the symptoms were relieved after leaving bed. There were no serious intraoperative complications and related postoperative adverse events in all patients. The JOA score for low back pain of all patients was significantly improved postoperatively at 1 month and the final follow‐up. All pedicle screws were clinically acceptable, and 41 (92.7%) were grade A and three (7.3%) were grade B on postoperative CT. At the final follow‐up, fusion was demonstrated in all patients without internal fixation failure and pseudoarthrosis.

**TABLE 2:** Radiological parameters.

Case no	Preoperative values/postoperative values								
	PT (°)	SS (°)	LL (°)	PI (°)	SVA (mm)	CVA (mm)	CA (°)	TK (°)	TLK (°)
1	30/22	30/38	31/45	60	128/55	40/20	18/6	16.2/23.3	9.8/9
2	3/13	42/32	25/43	45	170/70	30/7	20/9	14.8/26	9.8/7.9
3	26/20	35/41	38/51	61	−15/0	23/2	19/7	19/23	8.2/5.5
4	33/25	17/25	35.6/40	50	−20/−10	40/5	30/8.9	38/30	9/3
5	18/20	43/41	40/50	61	20/15	5/5	20/6	28/25	8/5
6	35/15	15/35	21/35.2	50	70/29.5	53.3/20.3	18.3/7	11/16	0/6

Abbreviations: CA, Cobb angle; CVA, coronal vertical axis; LL, lumbar lordosis; PI, pelvic incidence; PT, pelvic tilt; SS, sacral slope; SVA, sagittal vertical axis; TK, thoracic kyphosis; TLK, thoracolumbar kyphosis.

## Discussion

### Main Findings of this Study

Oblique lumbar interbody fusion is part of the stepped minimally invasive surgical strategies in the treatment of ADS, suitable for ADS patients who have the potential for interbody distraction and indirect decompression.^4^ Nonetheless, traditional OLIF technology often needs repositioning for one stage or staged posterior fixation for ADS, and the surgical steps are relatively complicated. OLIF 360 under navigation can perform OLIF and PPSF in a single lateral decubitus position simultaneously, and showed encouraging surgical outcomes and good spinal imbalance correction in treating modified MISDEF type II ADS patients in this study. However, care should be taken regarding patient selection and the surgical details of the OLIF procedure and PPSF under navigation.

### Indication for OLIF 360 in adult degenerative scoliosis

According to the MISDEF algorithm, patients classified as type 3 have fused or rigid spines, and the curve correction or lumbar lordosis remodeling effect of OLIF is limited. In these patients, a second‐stage posterior surgery and osteotomy are required.^11^ In ADS patients with modified MISDEF type 2, the OLIF procedure of multi‐level decompression and fusion with fixation might satisfy surgical demand, without the need for posterior osteotomy.^11^ In these patients, OLIF 360 technology simplifies surgical procedures, with OLIF and PPSF performed simultaneously in a single lateral decubitus position without repositioning.

Adult degenerative scoliosis is often complicated by moderate‐to‐severe spinal canal stenosis. Strict inclusion criteria are the key to achieve a good indirect decompression effect, and the disc distraction potential needs to be fully evaluated before the OLIF 360 procedure. Liu reported that OLIF with PPSF could achieve satisfactory clinical and radiographic effects in severe lumbar stenosis patients, but patients with bone fusion of the facet joint or severely calcified intervertebral discs were excluded.^15^ Shimizu excluded patients with extra‐ligamentous disc herniation, locked facets, and stenosis due to bony compression in an OLIF group, and the surgical and radiographic outcomes were better in an OLIF group than a conventional posterior fusion group.^5^ In this study, in the preoperative imaging evaluation, there were no signs of fusion of the facet joint and no bony narrowing of spinal canal. If disc spaces were rigid with lateral and anterior osteophyte bridges in patients with ADS, the osteophyte bridges should be released intraoperatively for sagittal and coronal imbalance correction. According to the preoperative symptom assessment, the patients’ symptoms were mainly caused by lumbar stenosis or mechanical low back pain, and the symptoms of lower limb were relieved completely after rest in bed. Although the patients’ situations were complicated by severe spinal stenosis in this study, all the patients achieved good indirect decompression with OLIF technology. The degree of spinal canal stenosis, mismatched lumbosacral pelvic parameters, and the sagittal and coronal imbalance were all significantly improved.

### Navigated OLIF 360 in adult degenerative scoliosis

The traditional OLIF technology requires one stage or staged posterior fixation in the treatment of ADS. Two operations need to be completed under two positions, and the surgical steps are relatively complicated. In OLIF 360, the two operations can be carried out simultaneously in decubitus position without mutual interference.^7^, ^8^, ^9^, ^10^ The operation steps are changed from series to parallel, greatly improving the efficiency of the operation. Meanwhile, in OLIF 360 single‐position workflow with navigation and O‐arm imaging, the instruments and implants are fully navigated, allowing for continuous visibility and precise placement with a real‐time image guidance mode, in which the accuracy and efficiency of PPSF and OLIF procedure are further improved.^8^, ^9^, ^10^

The concept of OLIF 360 is not novel and has been used in treating degenerative spinal instability. Sardhara reported that navigational‐assisted OLIF decreased the duration of surgery and showed encouraging early surgical outcomes, once the surgeon gains sufficient experience and becomes well‐versed at the procedure.^8^ Tan^16^ and Cheng^17^ found that single‐position OLIF combined with PPSF is safe and effective, and it has the advantages of a shorter operation time and less intraoperative blood loss. This study is the first to describe this technique for treating ADS. Different from OLIF 360 performed at one level, the PPSF and OLIF procedures were both performed from cephalic to caudal segments in ADS because navigational accuracy will decrease if the cage is placed in the caudal segment first.

There are many advantages of navigated OLIF in treating ADS. All procedures from the localization of intervertebral space to the placement of the cage are navigated, and repeated fluoroscopies are avoided.^8^, ^18^ The procedure of penetrating the contralateral annulus fibrosus and the cage placement are more accurate, especially in ADS patients with osteophytes or bone bridges on the lateral or contralateral side of the intervertebral space. Moreover, using navigation‐assisted OLIF, it is easier to determine the direction of the intervertebral space for further disc preparation in ADS, reducing the incidence of intraoperative complications such as poor positioning of the cage, end plate injury, or fracture. In this situation, we believe navigation‐assisted endplate preparation and accurate cage placement might prevent long‐term cage sinking and pseudarthrosis in ADS. Meanwhile, effectively releasing the osteophytes and fibrous anulus under navigation is important for the restoration of disc height, the recovery of lumbar lordosis, and the correction of coronal and sagittal imbalance.

Percutaneous freehand screw placement using C‐arm assistance is challenging in an unfamiliar position of OLIF 360.^17^ Robotic and navigation assistance have been used to improve the accuracy of PPSF. Budu^19^ reported that navigation can play an important role in patient safety during the surgeon's learning curve, which improves the accuracy of screw placement and reduces complications, especially in the hands of less experienced surgeons. Diaz‐Aguilar^7^ found that PPSF with robotic guidance resulted in greater accuracy (95%) in the decubitus position. In this study, all screw placements were clinically acceptable, and we believe that navigated percutaneous screw placement provides high screw insertion accuracy in the lateral decubitus position. Furthermore, OLIF 360 avoided the repeated fluoroscopic adjustment due to a different intervertebral space tilt in ADS during the operation, which significantly decreased the radiation exposure for the surgeon and operating room staff. The more complex the lumbar alignment restoration and PPSF procedure in ADS was, the better the operators’ experience was in the navigation‐assisted OLIF 360 technique.

In this study, no patient suffered from surgical site infection. It is crucial to ensure strict sterile manipulation during surgery, especially placing the pedicle screw in the lateral decubitus position. Moreover, PPSF on the lower side might be difficult and is influenced by the curve of lumbar scoliosis and the rotation of the vertebra in ADS. In this situation, the operating table can be rotated by an appropriate angle to neutralize the rotation and provide enough space for PPSF, and navigational accuracy is unaffected.

### Limitations and Clinical Prospects

The limitations of this study include its retrospective, single‐center design, the small sample size, and the absence of a control group, which restrict the generalizability and statistical power of the findings. Regarding fusion level, OLIF 51 is more difficult than OLIF 25 due to the vascular anatomy, and OLIF 51 is not included in this study. The OLIF technique involves a slight learning curve problem for surgeons. Furthermore, fused or rigid ADS with posterior osteotomy is required, and patients with radicular pain not relieved by bed rest are not suitable for OLIF 360. Further prospective research and increased sample sizes are needed to confirm the values of navigated OLIF 360 in ADS.

## Conclusion

We reported on a case series and described navigated OLIF 360 in treating ADS patients. Navigated OLIF 360 workflow allows continuous visibility and precise implant placement with real‐time image guidance in a single position, which significantly improves the accuracy and efficiency of the PPSF and OLIF procedures. Moreover, navigation‐assisted OLIF 360 has shown encouraging surgical outcomes with good spinal imbalance correction and indirect decompression.

## Conflict of Interest Statement

The authors do not have any possible conflicts of interest.

## Ethics Statement

This study was approved by the ethics committee of the Affiliated Hospital of Qingdao University. Written informed consent was obtained from the patient.

## Author Contribution

Conceptualization: YW and XM; Methodology: YW, CS, and SH; Formal analysis: SH and ZG; Writing‐original draft preparation: YW; Resources and Data curation: SH and CS; Supervision: YW and XM.
